# First time β-farnesene production by the versatile bacterium *Cupriavidus necator*

**DOI:** 10.1186/s12934-021-01562-x

**Published:** 2021-04-26

**Authors:** Sofia Milker, Dirk Holtmann

**Affiliations:** 1 Industrial Biotechnology, DECHEMA Research Institute, Theodor-Heuss-Allee 25, 60486 Frankfurt, Germany; 2 Institute of Bioprocess Engineering and Pharmaceutical Technology, Technische Hochschule Mittelhessen, Wiesenstr. 14, 35390 Gießen, Germany; 3 Fraunhofer Institute for Molecular Biology and Applied Ecology (IME), Research Division Bioresources, Ohlebergsweg 12, 35392 Giessen, Germany

**Keywords:** β-farnesene, Terpene production, *C. necator*, Sustainable economy, Process design

## Abstract

**Background:**

Terpenes are remarkably diverse natural structures, which can be formed *via* two different pathways leading to two common intermediates. Among those, sesquiterpenes represent a variety of industrially relevant products. One important industrially produced product is β-farnesene as a precursor for a jet fuel additive. So far, microbial terpene production has been mostly limited to known production hosts, which are only able to grow on heterotrophic substrates.

**Results:**

In this paper, we for the first time describe β-farnesene production by the versatile bacterial host *Cupriavidus necator* on fructose, which is known to grow hetero- and autotrophically and even in bioelectrochemical systems. We were able to show a growth-dependent production of β-farnesene by expressing the β-farnesene synthase from *Artemisia annua* in *C. necator* H16 PHB^-^4. Additionally, we performed a scale-up in a parallel reactor system with production titers of 26.3 ± 1.3 µM β-farnesene with a fed-batch process.

**Conclusions:**

The β-farnesene production titers reported in this paper are not in the same range as titers published with known heterotrophic producers *E. coli* or *S. cerevisiae*. However, this proof-of-principle study with *C. necator* as production host opens new synthesis routes toward a sustainable economy and leaves room for further optimizations, which have been already performed with the known production strains.

**Supplementary Information:**

The online version contains supplementary material available at 10.1186/s12934-021-01562-x.

## Background

Terpenoids are the largest group of natural products with substantial structural diversity. Among the terpenoids, sesquiterpenes (C_15_ molecules) are the largest subgroup, with more than 7000 individual compounds identified [1, 2]. The diversity of terpenes is derived from only two different precursor molecules isopentenyl pyrophosphate (IPP) and dimethylallyl pyrophosphate (DMAPP). IPP and DMAPP can be generated by one of two pathways: the mevalonate pathway (MVA), which is ubiquitous to eukaryotes and can also be found in archaea and some Gram-positive bacteria [3], or the methylerythritol-4-phosphate (MEP) pathway, found in some eukaryotes and in most bacteria, as in *Cupriavidus necator* [4]. The natural ability to provide precursors for terpene production [5] as well as its fast growth with high cell densities [6, 7] makes *C. necator* an attractive host organism to produce different industrially relevant molecules, e.g. terpenes. The sesquiterpenes derive from farnesyl pyrophosphate (FPP), which is formed by a reaction of one molecule DMAPP and two molecules of IPP. From FPP, the addition of a terpene synthase results in a one-step enzymatic transformation to the desired natural sesquiterpene. One of those sesquiterpenes is β-farnesene, a terpene converted from FPP by β-farnesene synthase. It has been identified as a precursor for the suitable jet fuel substitute farnesane (2,6,10-trimethyl dodecane) and is therefore of special interest [8]. Farnesane is formed by a complete hydrogenation of the β-farnesene C=C double bonds. So far, production is performed by the company Amyris Biotechnologies in a fermentation process in which genetically engineered yeast is cultivated on sugar cane syrup to produce the terpene farnesene, which is then subsequently converted to farnesane [9]. A production of β-farnesene and therefore farnesane from renewable sources which are not in competition with the food industry would be an attractive contribution to a carbon-neutral economy. A few publications already dealt with β-farnesene production and optimization of expression of the MVA pathway for a balanced precursor production in *E. coli* [10, 11] and reduction of side reactions in *Saccharomyces cerevisiae* [12]. However, all so far applied microorganisms for β-farnesene production were only able to consume heterotrophic substrates. Apart from the advantage of naturally expressing the MEP pathway for terpene synthesis, *Cupriavidus necator* can grow on a variety of substrates; heterotrophic carbon sources like fructose or glycerol are as well metabolized as lithoautotrophic substrates CO_2_ and H_2_. Two hydrogenases catalyze the oxidation of H_2_—one membrane bound hydrogenase transfers electrons into the electron transport chain, and one cytosolic hydrogenase generates reducing power by NADH regeneration for CO_2_ fixation. The growth on CO_2_ is mediated by the Calvin-Benson-Bassham cycle [13]. This autotrophic growth is also the basis for *C. necator*’s ability to grow in electrochemical systems, where H_2_ and O_2_ are produced at the electrodes by water electrolysis and CO_2_ is fed to the system [14]. In this work, we focus on an expression of a β-farnesene synthase in *C. necator* H16 PHB^−^4 to provide a proof-of-principle for β-farnesene production on lab scale in a controlled bioprocess, which later could be switched to an autotrophic or a bioelectrochemical process.

## Results and discussion

### Production of β-farnesene in *C. necator* in shake flasks

A vector pBBR1c_farn with β-farnesene synthase from *Artemisia annua*, which has previously been shown to functionally express in *E. coli* [15], was constructed from pBBR1c-RFP with the primers shown in Additional file 1. After transformation into *C. necator*, the β-farnesene production was assessed in a shake flask experiment in 20 mL minimal medium as aqueous phase. After induction at OD of 0.6 with L-arabinose, 5 mL n-dodecane were added for *in situ* product removal (ISPR), leading to a final total volume of 25 mL.

**Fig. 1:**
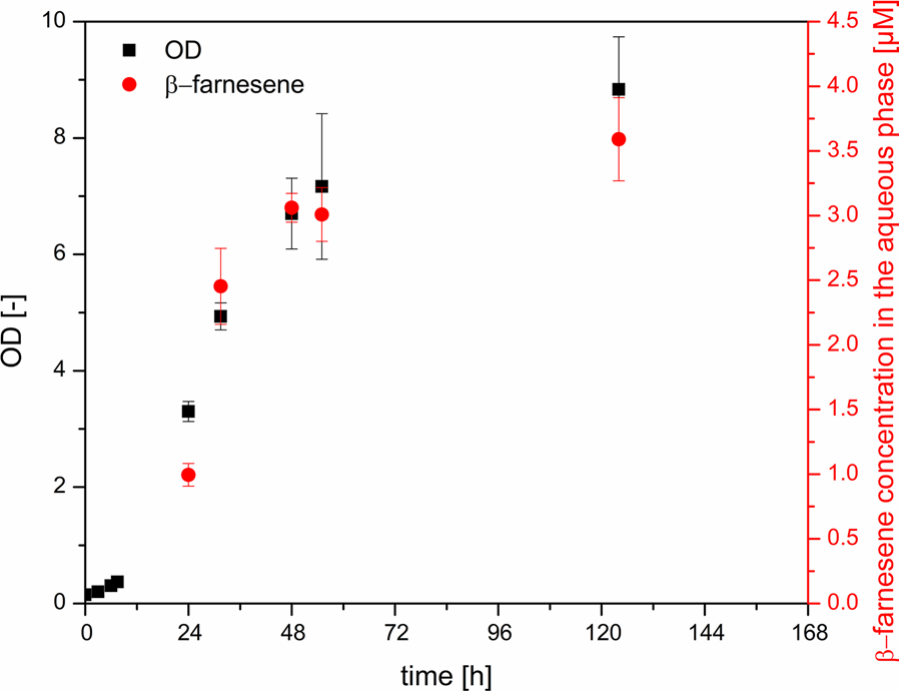
Production of β-farnesene by *Cupriavidus necator* pBBR1c_farn. Shake flask experiments in minimal medium (n = 3). Samples for β-farnesene were taken from the n-dodecane phase and related to the aqueous phase. Induction and n-dodecane addition were performed after 7.5 h of cultivation

**Fig. 2:**
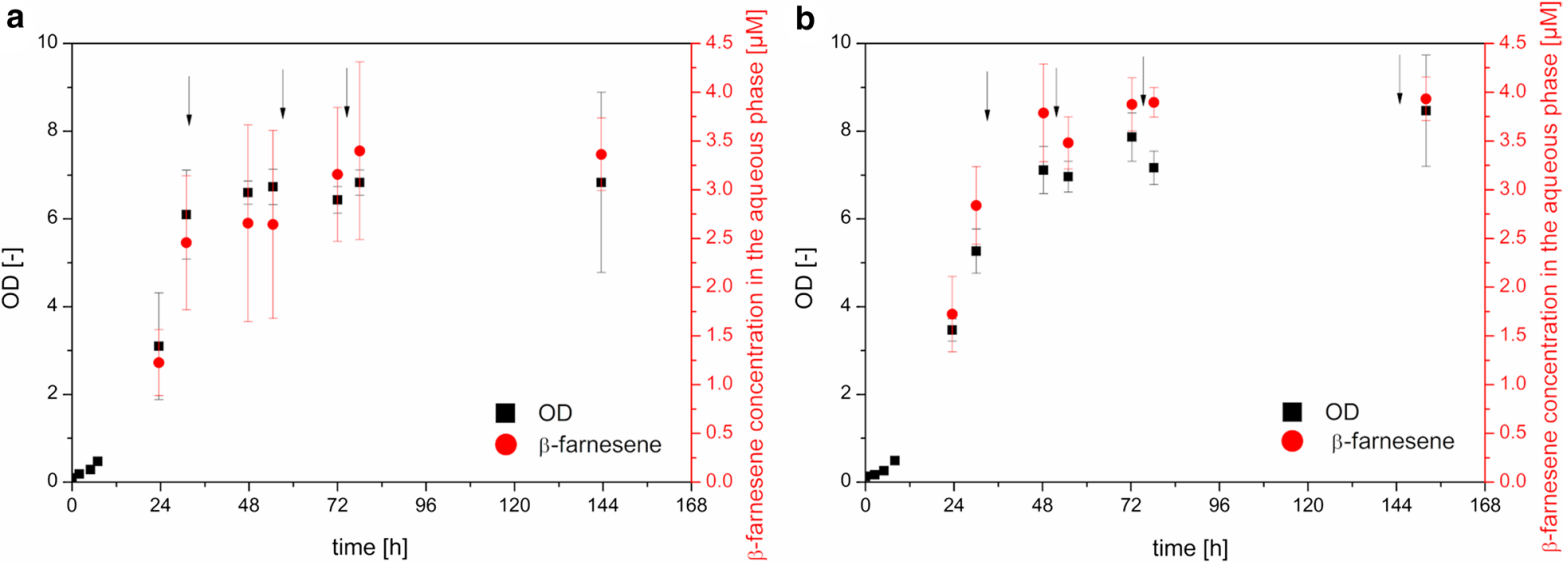
Influence of additional fructose and ammonium on β-farnesene production by C. necator pBBR1c_farn. **a** Spiking with fructose. **b** Spiking with fructose and ammonium sulfate in the stationary phase. Shake flask experiments in minimal medium (n = 3). Arrows indicate spiking of the respective substances. Samples for β-farnesene were taken from the n-dodecane phase and related to the aqueous phase. Induction and n-dodecane addition were performed after 7.5 h of cultivation

Figure 1 shows the typical *C. necator* biomass formation with induction and n-dodecane addition after 7.5 h of cultivation. The production started immediately after induction and reached a concentration of 1 µM after 24 h (total time). β-farnesene titers increased with biomass formation and led to constant titers in the stationary growth phase. The final OD value of 8.8 ± 0.9 and a β-farnesene concentration in the aqueous phase of 3.5 ± 0.32 µM (0.188 ± 0.017 mg gCDW^− 1^) were reached. To ensure, that the production was indeed growth-dependent, spiking experiments were performed. In the first set of experiments, only fructose (4 g L^− 1^) was spiked to the medium in the early stationary phase (Fig. 2a). In the second set of experiments, fructose (4 g L^− 1^) and ammonium sulfate (1.8 g L^− 1^) were spiked in the early stationary phase to see if the concentration of the carbon source or the overall cell growth limits the production (Fig. 2b).

Spiking with fructose alone did not increase β-farnesene titers compared to the simple batch process in the shaking flask with titers of 3.3 ± 0.4 µM in the aqueous phase. The OD was also comparable with a value of 6.8 ± 2.1. In contrast, when spiked with fructose and ammonium sulfate, the βfarnesene production titers increased to 3.9 ± 0.2 µM with a final OD of 8.6 ± 1.4. However, an unpaired t-test revealed that the difference for the OD formation is not significant and for the β-farnesene production likely not significant. However, we wanted to increase the amount of biomass in the reaction to produce more β-farnesene titers and we therefore aimed for addition of fructose and ammonium as a fed-batch process. Certainly, a detailed media optimization and an improved feeding protocol can lead to a further increase in β-farnesene titer and a deeper understanding of *C. necator* as terpenoid producer.

As a subsequent step, the plasmid with the β-farnesene synthase pBBR1c_farn was combined with the plasmid pKRTK3, a derivative of the previously published plasmid for α-humulene production in *C. necator* [16]. The pKRTK3 plasmid contains all the components of the mevalonate pathway, the IPP isomerase and the FPP synthase except for the α-humulene synthase. We wanted to increase the overall flow toward the precursors for β-farnesene synthesis. Since the native MEP pathway in *C. necator* is regulated, our rationale was to add an unregulated second pathway to increase the amount of precursors. Combined with the β-farnesene synthase, the pKRTK3 plasmid should, in theory, lead to higher β-farnesene titers. Therefore, the pBBR1c_farn plasmid and the pKRTK3 plasmid were both transformed into the same *C. necator* H16 PHB^−^4 strain. The resulting strain *C. necator* pBBR1c_farn pKRTK3 strain (pathway scheme in Additional file 1: Figure S2) was cultivated as in the previous experiment with addition of fructose in the stationary phase, since the production *via* the heterologously expressed MVA should not be growth-dependent.

The double plasmid system with additional MVA expression led to a decrease in β-farnesene production titers by two thirds compared to the simple expression of the β-farnesene synthase with final titers of 0.9 ± 0.2 µM vs. the previously reported 3.3 ± 0.4 µM in the aqueous phase with the same experimental set-up (Additional file 1: Figure S3). Even though high OD values with 6.4 ± 0.3 were observed after 24 h of cultivation, the biomass rapidly decreased afterwards. This seems to be a stress related cells lysis which occurred shortly before the stationary phase is reached, leading to a decrease in optical density. This stress is likely related to the use of two different expression vectors with two distinct antibiotics resistances. The metabolic burden of maintain the plasmids and expressing the coded genes of interest is higher for two plasmids compared to one plasmid [17, 18]. The two-plasmid system did not show any advantages over the expressing strain with only β-farnesene synthase inserted.

Since the two-plasmid system is not stable and therefore cannot be used for a large-scale production, the one plasmid system *C. necator* pBBR1c_farn was chosen for a scale-up.

### Production of β-farnesene in a fed‐batch system

As fermentation process, a fed-batch was chosen since it is crucial to a high biomass concentration with *C. necator* pBBR1c_farn to increase β-farnesene titers, as has been showed in previous shake flask experiments. The production was performed in a DASGIP parallel reactor system (n = 4). The feed contained fructose and nitrogen.

**Fig. 3:**
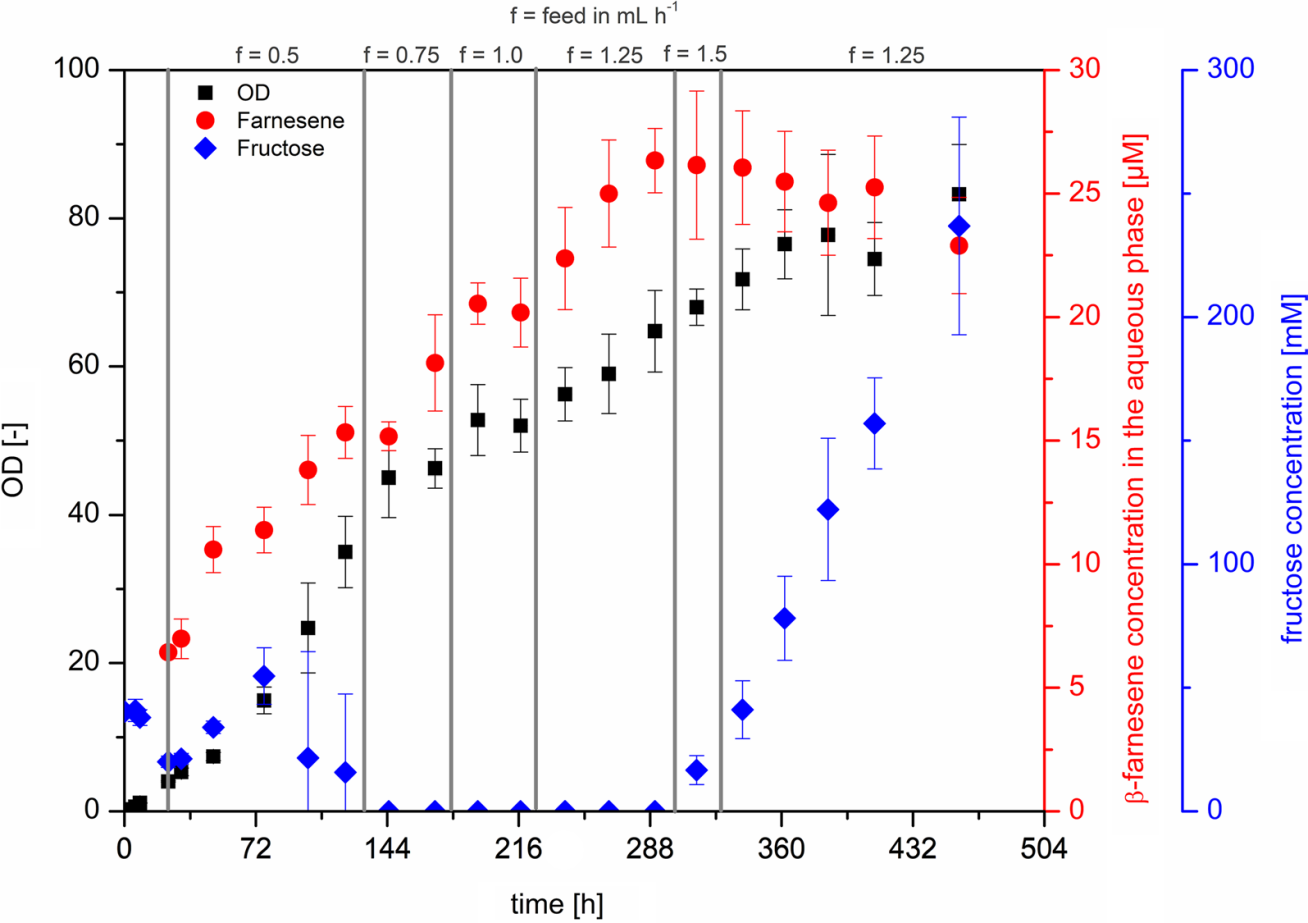
Fed-batch fermentation process of C. necator pBBR1c_farn in a DASGIP parallel reactor system. Cultivation in minimal medium with initial volume of 300 mL at 30 °C and a final volume of 900 mL. pH regulated to 6.8 with 2 M NaOH. Aeration and stirring regulated by a regulation cascade with a DO setpoint at 30%. Induction after 9 h with 0.2% L-arabinose and addition of 100 mL n-dodecane for ISPR. Feeding start after 24.5 with varying feed rates. Performed in replicates: n = 4. Samples for β-farnesene were taken from the n-dodecane phase and related to the aqueous phase. OTR and CTR data can be found in Additional file 1

After inoculation, the process was first run as a batch with the corresponding decrease in fructose and an increase in optical density (Fig. 3). After the first 9 h, the β-farnesene synthase expression was induced with L-arabinose, and n-dodecane was added to the reactor for *in situ* product removal. After 24.5 h of cultivation the fedbatch phase was started with a feed rate of f = 0.5 mL h^− 1^. During this feed rate, the biomass production increased with a growth rate of µ = 0.33 ± 0.04 h^− 1^ was then limited by the fructose concentration after 144 h of cultivation. The feed rate was increased f = 0.75 mL h^− 1^ and shortly after to f = 1.0 and f = 1.25 mL h^− 1^ to increase the production velocity. Until the feed rate was set to f = 1.5 mL h^− 1^ after 291 h of cultivation, the process was limited by the feed rate. With a feed rate of f = 1.5 mL h^− 1^, the *C. necator* cells were not able to reach the required growth rate to consume all the fructose fed to the reactor. This led to a change in fructose metabolism. If there was no change in the metabolism, the fructose would have been metabolized as it has been before. Even though the feed was reduced to f = 1.25 mL h^− 1^ after one fructose sample after 319 h of cultivation, the system could not be stabilized. The biomass formation was stable until the experiment was stopped after 457 h. However, the production related to the aqueous phase remained constant. This can be explained by the decrease in production and dilution by the feed since the concentration in the organic phase was slightly increasing until the end of the experiment. The reactors did not show any oxygen limitation at any time during the process (Additional file 1: Figure S4).

The production was coupled to biomass formation, as already observed in the shakeflask experiments. The highest concentration of β-farnesene in the aqueous phase was detected after 290 h with 26.3 ± 1.3 µM (0.20 ± 0.01 mg gCDW^− 1^; corresponding to 4.12 mg in total). To the best of our knowledge, this is the first time that β-farnesene production was shown in *C. necator*. So far, a *S. cerevisiae* strain expressing the same β-farnesene synthase from *A. annua* was able to produce 592 µM (121 mg L^− 1^) in a fed-batch reactor experiment with a RQ-controlled feed [12]. The strain was modified to downregulate a competing squalene synthase and had two knockouts to inhibit competing farnesol production. In the same publication, a strain which additionally had an upregulation of FPP-synthase and a deletion of an NADPH-dependent glutamate synthase, while having an upregulated NADH-dependent glutamate synthase and expressing a farnesene synthase from *Malus domestica* produced 827 µM (169 mg L^− 1^) farnesene. In *E. coli*, a fully balanced heterologously-expressed MVA pathway with a β-farnesene synthase from *A. annua* was able to produce 42.8 mM (8.74 g L^− 1^) β-farnesene [10]. Even though the titers reported in this manuscript cannot compete with productions in *E. coli* and *S. cerevisiae*, this system has still an enormous potential for optimization, which have been already performed with those strains as introducing a balanced heterologously-expressed MVA pathway, knockouts of competing reactions and upregulation of desired native reactions. However, with *C. necator*, a more ecologically friendly process based on H_2_/CO_2_ or CO_2_ and current can be developed, which cannot be easily implemented with the existing β-farnesene producers.

## Conclusions

The expression of β-farnesene synthase led to a first-time production of β-farnesene in *Cupriavidus necator* H16 PHB^-^4. As observed in shake-flask experiments, the production was growth-dependent. This observation led to the development of a fed-batch process, producing titers of 26.3 ± 1.3 µM β-farnesene with the over-expression of only one enzyme. These values can so far not compete with already established recombinant β-farnesene producers as *E. coli* and *S. cerevisiae*. Nevertheless, *C. necator* is a promising new producing strain with a broader substrate scope (organic acids and waste streams like glycerol), including autotrophic substrates (mixture of H_2_, O_2_ and CO_2_) and the possibility of being cultivated in bioelectrochemical systems with and CO_2_ current. Especially for novel terpenoid production hosts such as *C. necator*, it will be essential to analyze the impact of recombinant terpenoid production on host metabolism and accordingly adapt and balance pathway expression and regulation [19]. Further improvements, like a construction of a one plasmid system with the combination of the balanced MVA pathway and β-farnesene synthase as previously shown for *E. coli* or upregulation of native enzymes and knockouts of competing reactions as shown for *S. cerevisiae* would improve yields. Additionally, it would revolutionize the synthetic fuel production, as β-farnesene is a precursor for farnesane, a certified drop-in substance for synthetic fuels.

## Methods

### Cultivation media

Lysogeny broth (LB) was used as complex medium for precultures, if not stated otherwise. LB was composed of (in g L^− 1^): tryptone 10, yeast extract 5, NaCl 10. Minimal medium (MM) for *C. necator* was composed of (in g L^− 1^): Na_2_HPO_4_ 2.895, NaH_2_PO_4_*2 H_2_O 3.06, K_2_SO_4_ 0.17, CaSO_4_*2 H_2_O 0.097, MgSO_4_*7 H_2_O 0.8, (NH_4_)_2_SO_4_ 1.886, FeSO_4_*7 H_2_O 0.05, trace elements 1:20,000. Trace elements stock was composed of the following ingredients (in g L^− 1^, in 0.05 M H_2_SO_4_): FeSO_4_*7 H_2_O 15, MnSO_4_* H_2_O 2.4, ZnSO_4_*7 H_2_O 2.4, CuSO_4_*5 H_2_O 0.48, Na_2_MoO_4_*2 H2O 1.8, Ni_2_SO_4_*6 H2O 1.5, CoSO_4_*7 H2O 4.02*10^− 2^. Fructose was added to the MM to a final concentration of 8 g L^− 1^ (44.4 mM) under heterotrophic conditions, if not stated otherwise. Antibiotic concentrations were 125 µg mL^− 1^ chloramphenicol (CAM, 250 mg mL^− 1^ stock in ethanol) and 15 µg mL^− 1^ tetracycline hydrochloride (Tc, 10 mg mL^− 1^ stock in water) for recombinant strains. L-arabinose for the pBBR1c_farn and L-rhamnose for the pKRTK3 plasmid were used as inducers at a final concentration of 11 mM (0.2 %) using a 20 % (w/v) stock solution for β-farnesene production. All basic media components were purchased from Sigma-Aldrich (Munich, Germany) or Carl Roth (Karlsruhe, Germany).

### Plasmid construction

The β-farnesene synthase from *Artemisia annua* [15] was introduced into the pBBR1c-RFP plasmid, exchanging the red fluorescent protein sequence [20] by method of Gibson [21]. The detailed description is given in Additional file 1. The pKRTK3 (pKRhum Δ ZSSI) construction was described elsewhere [16].

### Transformation of plasmids into *E. coli* and *C. necator*

*E. coli* strains were made chemically competent for plasmid uptake by a standard protocol [22]. *E. coli* DH5α cells were transformed with 1–4 µL Gibson assembly product by heat shock (30 s at 42 °C) and aliquots were plated on LB agar supplemented with the selective marker. The verification of correct constructs was performed by sequencing.

Plasmids were transferred to the recipient *C. necator* H16 PHB^−^4 by electroporation. The *C. necator* strain was grown as main culture in LB medium until an OD of 0.6. The cells were centrifuged and washed three times with cold and sterile 10% (v/v) glycerol solution with centrifugation steps in-between. After centrifugation, the pellet was resuspended the pellet in a 1/100 of the original volume of 10% (v/v) glycerol solution and aliquot 50–100 µL per pre-cooled 1.5 mL reaction tube. The cells were stored at − 80 °C until electroporation.

For the electroporation, the electroporation cuvettes (2 mm width) were cooled on ice and at least 2 µl DNA (50–100 ng/µL) were added. The cuvette was pulsed with 2500 V, 25 µF und 400 Ω and 900 µL LB Medium were immediately added. The mixture was transferred to a 1.5 mL reaction tube and the cells were regenerated at 30 °C for 3 h with shaking of 700 rpm (ThermoMixer, Eppendorf, Hamburg, Germany). The cells were centrifugated for one minute at 16000×*g*, 700 µL of the supernatant were removed and the pellet was resuspended in the remaining supernatant and immediately plated on an agar plate with the appropriate selection marker.

### Heterotrophic cultivation in shake flasks

Strains were cultivated at 30 and 37 °C for *C. necator* and *E. coli*, respectively. Liquid cultures were shaken at 180 rpm in the incubation shakers Minitron or Ecotron (Infors AG, Bottmingen, Switzerland) with a deflection of 25 mm. For β-farnesene production experiments, the cells were cultivated in 100 mL non-baffled shake flasks with 20 mL minimal medium induced at an OD of 0.6 and 5 mL n-dodecane (20% of the total volume) were added for *in situ* product removal (ISPR). The samples for β-farnesene production were taken from the organic n-dodecane phase and analyzed with LC-MS/MS. An unpaired t-test for OD measurements and β-farnesene titers was performed between fructose and fructose/ammonium spiking cultures with confidence intervals of 95%.

### Quantification of fructose in the supernatant with HPLC

Fructose concentration was quantified by HPLC (SCL 10-A, Shimadzu Deutschland GmbH, Duisburg, Germany) equipped with the refractive index detector RID-10 A and provided with LabSolutions, version 5.57 software. A 30 cm Rezex ROA organic acid H+ (8%) analytical column was used (Phenomenex Inc., California, USA). Elution was preceded with 5 mM H_2_SO_4_ at a flow of 0.6 mL/min and 30 °C [23].

### Quantification of β-farnesene production via LC-MS/MS

β-farnesene was quantified from the organic n-dodecane phase with an LC-MS/MS system consisting of a triple quadrupole mass spectrometer (LCMS-8040, Shimadzu Deutschland GmbH, Duisburg, Germany) equipped with an atmospheric pressure ionization source (APCI; Shimadzu Deutschland GmbH, Duisburg, Germany) and an associated uHPLC system (Nexera 30 series, Shimadzu Deutschland GmbH, Duisburg, Germany). Mass spectrometric measurements were performed without preceding chromatographic separation. Methanol was used as the mobile phase at a flow rate of 0.25 mL min^− 1^. APCI was used for positive ionization. Parameters for APCI were the following: nebulizing gas flow 3 L min^− 1^, drying gas flow 5 L min^− 1^, interface temperature 350 °C, desolvation line temperature 200 °C, heat block temperature 200 °C and interface voltage 4.5 kV.

The mass spectrometer was run in multiple reaction monitoring (MRM) mode [16, 24] for the selective quantification of β-farnesene (MRM (+) m/z 205.25 → m/z 149, m/z 205.25 → m/z 107, m/z 205.25 → m/z 135) with 50 µM caffeine in acetone as internal standard (MRM(+) m/z 194.80 → m/z 138, m/z 194.80 → m/z 42, m/z 194.80 → m/z 110). MRM method parameters (collision energies, dwell times and exact m/z values) were automatically optimized with the software (LabSolutions Version 5.95, Shimadzu Deutschland GmbH, Duisburg, Germany).

Samples and external standards were dissolved in acetone with caffeine as internal standard (1:10) with 90 µL of acetone with 50 µM caffeine being mixed with 10 µL of n-dodecane extract. The extract was not dried since water is completely immiscible with n-dodecane. Quantification was done using a linear calibration curve with external standards. The product concentrations given are related to the producing, aqueous phase.

### Heterotrophic fed‐batch fermentation in DASGIP® parallel fermentation system

*C. necator* pBBR1c_farn was cultivated in a SR1000ODLL bioreactor system connected to the sensor module pH4pO4 for pH and dissolved oxygen (DO) monitoring and control, TC4SC4 module for agitation and temperature control, exhaust gas analyzer GA4, multi pump module MP8, and compressed air supplier MX4/4 (DASGIP, Jülich, Germany). For pH control, the pH was maintained at 6.8 with a 2 M NaOH solution. The applied feeding solution composition was as follows: 422.3 g L^− 1^ fructose, 117.5 g L^− 1^ (NH_4_)_2_SO_4_ and 1x addition of trace elements as in the regular minimal medium. The reactors were placed into a BioBlock to control the temperature at 30 °C. The regulation of the process was performed with DASGIP control, v4.5. The initial settings for gassing were set to 6 standard liter per hour (sL h^− 1^) and the initial agitation speed was set to 400 rpm. The agitation and gassing were regulated to keep the DO signal at 30 %. Initial liquid volume was set to 0.4 L. A preculture grown over night in minimal medium was used for inoculation to an OD of 0.25.

Cultures were induced at OD of 1.1 after 9 h with 0.2% L-arabinose. At induction time, 20% n-dodecane (100 mL) were added to the culture through a port to remove β-farnesene *in situ*. The feed was started after 24.5 h with the initial flow rate of 0.5 mL h^− 1^ and was increased throughout the fermentation until a maximal feed of 1.5 mL h^− 1^. Increase in biomass and product formation and concentration of carbon and nitrogen source were followed over time. Optical density (OD) was measured at 600 nm using Biowave Cell Density Meter CO8000 (Biochrom WPA, Cambridge, England). The biomass OD dependency used according to:

- Biomass (g L^− 1^) = 0.43 × OD [16].

## Supplementary Information

**Additional file 1.** The construction of the plasmids to produce farnesene is described in detail. In addition, the MVA pathway (plasmid pKRTK3) coupled with the β-farnesene synthase (plasmid pBBR1c_farn) expression is presented. The calculations of the oxygen and carbon dioxide transfer rates (OTR/CTR) via the gas-balancing method is shown. Furthermore, the β-farnesene calibration with caffeine as internal standard is described.

## Data Availability

All data generated or analyzed during this study are included in this published article and its supplementary information files.
