# Supplementary material for: First time β-farnesene production by the versatile bacterium Cupriavidus necator
Source: Microb Cell Fact. 2021 Apr 26;20:89. doi: 10.1186/s12934-021-01562-x (PMC8074451; doi:10.1186/s12934-021-01562-x)
Supplement: Supplementary file 1 — Additional file 1. The construction of the plasmids to produce farnesene is described in detail. In addition, the MVA pathway (plasmid pKRTK3) coupled with the β-farnesene synthase (plasmid pBBR1c_farn) expression is presented. The calculations of the oxygen and carbon dioxide transfer rates (OTR/CTR) via the gas-balancing method is shown. Furthermore, the β-farnesene calibration with caffeine as internal standard is described. [file 12934_2021_1562_MOESM1_ESM.docx]

**First time β-farnesene production with the versatile bacterium *Cupriavidus necator***

Sofia Milker and Dirk Holtmann

**Supplementary Information**

**Construction of the pBBR1c_farn vector**

For the construction of the pBBR1c_farn vector, the previously published pBBR1c_RFP vector was used as a backbone. It was amplified with the primers pBBR1c_backbone_fwd/pBBR1c_backbone_rev (table S1). The insert was ordered from a gene synthesis company (BioCat, Heidelberg, Germany) and amplified with Gibson primers Farn_pBBR1c_fwd/ Farn_pBBR1c_rev with overhangs of 20 bp.

**Table S1:Primer for pBBR1c_farn vector construction**

| **Primer name** | **Primer sequence** |
| --- | --- |
| pBBR1c_backbone_fwd | GGATCCAAACTCGAGTAAGGATCTCCAG |
| pBBR1c_backbone_rev | ATGTATATCTCCTTCTTAAAAGATCTTTTGAATTCCCAAAAAAACGG |
| Farn_pBBR1c_fwd | TTTGGGAATTCAAAAGATCTTTTAAGAAGGAGATATACATATGTCGACCCTGCCGATCTC |
| Farn_pBBR1c_rev | TTTATTTGATGCCTGGAGATCCTTACTCGAGTTTGGATCCTCACACCACCATCGGGTGC |

Both PCR products were amplified from an agarose gel electrophoresis with a DNA gel extraction kit (Zymoresearch, Freiburg, Germany) and ligated with the Gibson cloning protocol [1] (Fig. S1).


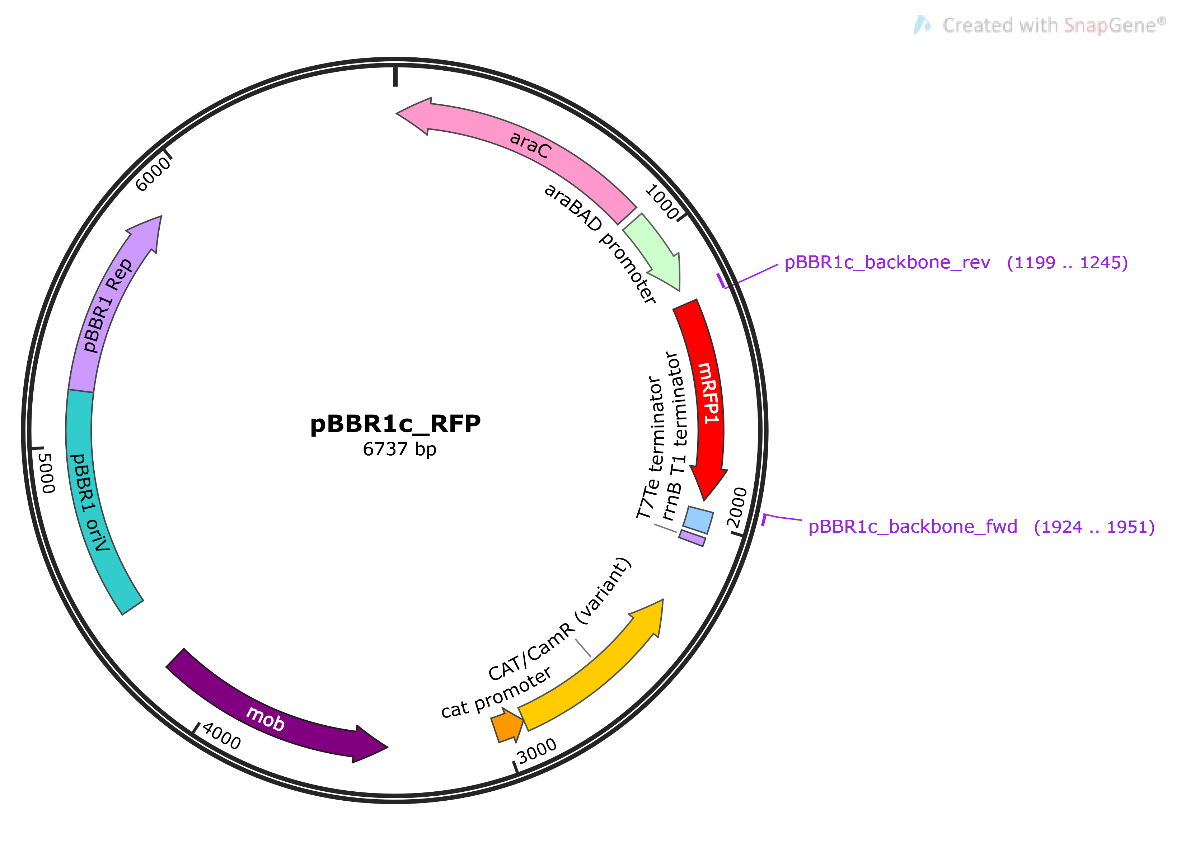

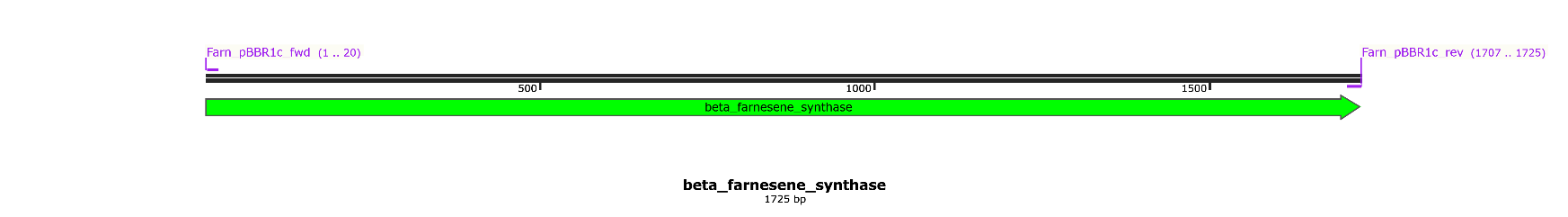

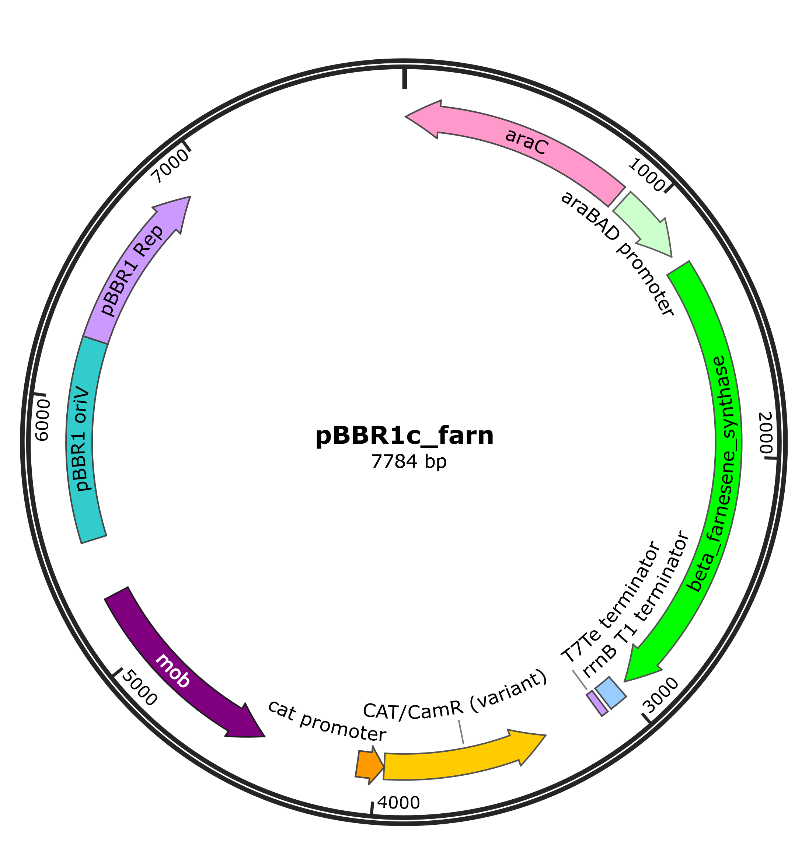


**Figure S1: Cloning Scheme for pBBR1c_farn vector construction**

**The desired MVA – β-farnesene synthase pathway**

**

**

**Figure S2: The desired MVA pathway (plasmid pKRTK3) coupled with the β-farnesene synthase (plasmid pBBR1c_farn) expression.**

**Production of β-farnesene with the *C. necator* H16 PHB^-^4 pBBR1c_farn pKRTK3 strain**





**Figure S3: β-farnesene production with C. necator pBBR1c_farn pKRTK3 strain.**

Shake flask experiments in minimal medium (n=3). Samples for β-farnesene were taken from the n-dodecane phase and related to the aqueous phase.

**Oxygen and carbon dioxide transfer rate (OTR/CTR) calculations via the gas-balancing method**

The oxygen transfer rate (OTR) and carbon dioxide transfer rate (CTR) were calculated according to the following equations: [2-4]

$$OTR= \frac{Q}{V}(C_{O_{2}}^{in}- C_{O_{2}}^{out})$$

$$CTR= \frac{Q}{V}(C_{{CO}_{2}}^{out}- C_{CO_{2}}^{in})$$

With Q being the oxygen gas flow, V being the volume of the bioreactor and C_in_ and C_out_ being the carbon and oxygen concentrations measured at bioreactor inlet and outlet.

Carbon dioxide transfer rate (CTR) and oxygen transfer rate (OTR) reveal insights into the biological activity by showing the carbon dioxide evolution and oxygen consumption. give the equations for the calculations of CTR and OTR, respectively. The liquid volume without n-dodecane was considered for the aeration rate. The respiratory quotient defines the relation between the oxygen consumed and carbon dioxide produced

$$OTR= \frac{q_{in}}{V_{mo}}(y_{O_{2}, in}- \frac{1-y_{O_{2}, in}-y_{{CO}_{2}, in}}{1-y_{O_{2}, out}-y_{{CO}_{2}, out}}y_{O_{2}, out})$$

$$CTR= \frac{q_{in}}{V_{mo}}(y_{{CO}_{2}, out}\frac{1-y_{O_{2}, in}-y_{{CO}_{2}, in}}{1-y_{O_{2}, out}-y_{{CO}_{2}, out}}-y_{CO_{2}, in})$$

| **parameter** | **description** | **unit** |
| --- | --- | --- |
| V_mo_ | molar gas volume at standard conditions | [L/mol] |
| q_in_ | Specific aeration rate at inlet | [vvm] |
| q_out_ | Specific aeration rate at outlet | [vvm] |
| y_x,in_ | X mole fraction in inlet gas | [mol/mol] |
| y_x,out_ | X mole fraction in outlet gas | [mol/mol] |

**OTR, CTR and RQ signals for the parallel reactor system**





**Figure S4: OTR, CTR and RQ signals for the reactors in the C. necator pBBR1c_farn fermentation.** A-D: reactors 1-4.

**β-farnesene calibration with caffeine as internal standard**

**

**

**Figure S5: exemplary β-farnesene calibration with caffeine as internal standard.** For every measured batch, the calibration was performed freshly, measured once before the measurement of the samples. One standard was measured between the samples to ensure a stable signal.

**References:**

1. Gibson DG, Young L, Chuang R-Y, Venter JC, Hutchison CA, Smith HO: **Enzymatic assembly of DNA molecules up to several hundred kilobases.** *Nat Methods* 2009, **6:**343-345.

2. Royce PN: **Effect of changes in the pH and carbon dioxide evolution rate on the measured respiratory quotient of fermentations.** *Biotechnol Bioeng* 1992, **40:**1129-1138.

3. Garcia-Ochoa F, Gomez E, Santos VE, Merchuk JC: **Oxygen uptake rate in microbial processes: An overview.** *Biochem Eng J* 2010, **49:**289-307.

4. Suresh S, Srivastava V, Mishra I: **Techniques for oxygen transfer measurement in bioreactors: a review.** *J Chem Technol Biotechnol* 2009, **84:**1091-1103.
